# Prevalence of Heritable Symbionts in Parisian Bedbugs (Hemiptera: Cimicidae)

**DOI:** 10.1111/1758-2229.70054

**Published:** 2025-01-29

**Authors:** Naciye Sena Cagatay, Mohammad Akhoundi, Arezki Izri, Sophie Brun, Gregory D. D. Hurst

**Affiliations:** ^1^ Institute of Infection, Veterinary and Ecological Sciences University of Liverpool Liverpool UK; ^2^ Parasitology‐Mycology Department, Avicenne Hospital, AP‐HP Sorbonne Paris Nord University Bobigny France; ^3^ Unité Des Virus Émergents (UVE: Aix‐Marseille Université‐IRD 190‐Inserm 1207‐IHU Méditerranée Infection) Marseille France

**Keywords:** *Ca.* Tisiphia, *Cimex*, *Symbiopectobacterium*, symbiosis, *Wolbachia*

## Abstract

Like many insects, the biology of bedbugs is impacted by a range of partner heritable microbes. Three maternally inherited symbionts are recognised: *Wolbachia* (an obligate partner), *Symbiopectobacterium purcellii* strain *Sy*Clec, and *Candidatus* Tisiphia sp. (facultative symbionts typically present in some but not all individuals). Past work had examined the presence of these heritable microbes from established laboratory lines, but not from broader field samples. We therefore deployed targeted endpoint PCR assays to determine the symbiont infection status for 50 bedbugs collected from 10 districts of Paris during the 2023 outbreak. All three symbionts were found to be broadly present across *Cimex lectularius* samples, with the *Symbiopectobacterium*‐*Candidatus* Tisiphia‐*Wolbachia* triple infection most commonly observed. A minority of individuals lacked either one or both facultative symbionts. Five mtDNA haplotypes were observed across the COI barcode region, and triple infections were found in all mtDNA haplotypes, indicating that symbiont infection is not a recent invasion event. We conclude that the Parisian bedbug outbreak was one in which the host's secondary symbionts were present at high‐frequency coinfections, and facultative symbionts are an important but uncharacterised component of bedbug populations.

## Graphical abstract

Bedbugs are known to host three symbionts: an obligate partner, *Wolbachia*, and two facultative symbionts, *Symbiopectobacterium* and *Candidatus* Tisiphia. However, the frequency of these symbionts in natural populations is unknown. We tested materials from the Paris 2023 bedbug outbreak and found that most *Cimex lectularius* individuals carry all three symbionts, with *Wolbachia* and *Symbiopectobacterium* double infections being the next most common. We conclude that research on bedbug biology in natural settings must consider the impact of the symbionts they carry.

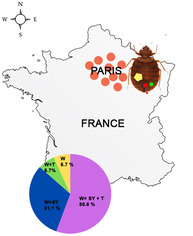

## 1 Introduction

Hemipteran insects have diverse and important symbioses with microbial partners. Symbionts contribute to processes of digestion, anabolic activities (supply of vitamins & amino acids) (Akman et al. 2002), protection against natural enemies (Gosalbes et al. 2010), degradation of insecticides (Su, Zhou, and Zhang 2013) and competence of their host for transmitting viral diseases (Kliot et al. 2014). In contrast, other symbiotic microbes exhibit pathogenic or parasitic characteristics. Parasitic interactions may involve maternal inheritance of the symbiont, leading to selection for sex ratio distortion (Hurst and Frost 2015).

The symbiotic relationships that insects form are crucial drivers of their biology. These interactions present new opportunities for controlling pest species and vector competence. For instance, targeting obligate symbionts can serve as an effective means to eliminate focal parasites, as seen in filariasis treatment where filarial infection is cured by selectively eradicating the worm's *Wolbachia* symbionts (Johnston et al. 2021). Additionally, modifying symbiont strains to alter viral transmission competence is widely applied in controlling Zika and dengue transmission from mosquito hosts (Ant et al. 2023).

In this study, we examine the symbiotic associations of bedbugs, which are global pests whose their blood‐feeding activity causes a significant nuisance, with particular reputational and financial impacts in the hospitality sector (Doggett et al. 2012). Outbreaks of bedbugs—where infestations become locally frequent—are commonly observed and attract considerable press coverage. For instance, outbreaks of the common bedbug (*Cimex lecturalis* Linnaeus, 1758) and the tropical bedbug ( *Cimex hemipterus* Fabricius, 1803) have occurred regularly in Paris, France, with a notable peak event in hospitality residences in 2023 (Chebbah et al. 2023; Brimblecombe, Mueller, and Querner 2024).

As obligate blood feeders, bedbug development and reproduction relies on the presence of an obligate *Wolbachia* symbiont that supplies B vitamins to the host. Aposymbiotic bedbugs exhibit slower nymphal development, reduced adult survivorship, smaller adult size, fewer eggs per female, and a lower hatch rate comparing to bedbugs that harbour *Wolbachia* (Hickin, Kakumanu, and Schal 2022; Kakumanu, Hickin, and Schal 2024). Bed bugs experimentally depleted of *Wolbachia* can only develop with B vitamin supplementation, and the *Wolbachia* genome was found to contain a biotin synthesis operon within it (Hosokawa et al. 2010; Nikoh et al. 2014).

Bedbugs additionally host two other heritable symbiont microbes, but these contrast with *Wolbachia* in not being required for the bedbug to develop and reproduce. They are therefore regarded as facultative symbionts, and indeed laboratory lines of bedbugs vary in the presence and frequency of these strains. The first recognised was a gammaproteobacterium that falls in the recently described species *Symbiopectobacterium purcellii* (Nadal‐Jimenez et al. 2022). Described initially by Hypša and Aksoy (1997) from a laboratory colony established in the USA, this microbe (henceforth *S. purcellii* strain *Sy*Clec) was later visualised as infecting the host ovaries alongside *Wolbachia* in Japanese bedbug strains (Hosokawa et al. 2010) and in the UK (Thongprem et al. 2020). Recent studies have observed *Symbiopectobacterium* in a variety of hemipteran insects, a member of the Hymenoptera (Tvedte et al. 2019), and in a nematode (Martinson et al. 2020). Subsequently, a *Ca*. Tisiphia amplicon was detected in *C. lectularius* (Pilgrim et al. 2021). Additional research confirmed the maternal inheritance of the *Ca*. Tisiphia symbiont in bedbugs and noted presence in 13 out of 21 laboratory strains originating from Europe and Africa (Thongprem et al. 2020). Infection with this symbiont was associated with a modest impact on host life history characters but with no evidence of reproductive parasitism (Thongprem et al. 2020). *Ca*. Tisiphia is a genus sister to *Rickettsia* that includes many invertebrate heritable microbes (Davison et al. 2022).

Because the biology of insects is defined in part by the presence and frequency of circulating symbionts, they represent an important factor to consider in outbreaks. Previous research on bedbug heritable microbes has primarily focussed on diverse established laboratory lines, rather than bedbugs in the field context. We therefore analysed the pattern of presence and diversity of heritable symbionts in bedbugs collected across Parisian localities during the 2023 outbreak. Within this study, we also sequenced the associated mtDNA of the bedbug host. mtDNA and symbionts are coinherited through the female line, and the diversity of mtDNA diversity is used to infer the recency of the spread of symbionts (Galtier et al. 2009). For instance, recent symbiont spread produces an associated sweep of the initially coinherited mtDNA haplotype and commonly reduces mtDNA to a single circulating haplotype (e.g., Hale and Hoffmann 1990). Conversely, the presence of mtDNA diversity in symbiont‐infected lineages implies a longer history of association (e.g., Dyer and Jaenike 2004). Thus, mtDNA analysis allows coarse inference of the recent history of selection on the symbiont.

## 2 Materials and Methods

### 2.1 Bed Bug Collection and Identification

Bed bug samples were collected from various infested locations in the Paris area (Table 1). All the specimens were labelled and kept at −20 °C for further molecular analysis.

**TABLE 1:** Collections sites of bedbugs in Paris in summer 2023.

Location	Accommodation type
District 9 of Paris	Private apartment
District 11 of Paris	Private apartment
District 18 of Paris	Private apartment
District 19 of Paris	Private apartment
District 20 of Paris	Private apartment
Bobigny	HLM (social Collective apartment)
La Courneuve	Refugee camp residence
Pantin	Private apartment
Montreuil	Retirement home
Aubervilliers	HLM (social collective apartment)

### 2.2 DNA Extraction, PCR Amplification and Sequencing

Bedbug DNA was extracted individually using Wizard Genomic DNA Purification kit (Promega, USA). The DNA was resuspended in 50 μL of molecular‐grade water and stored at −20 °C for future use. Amplification of the COI gene was initially assessed as a means of quality control by conventional PCR assay using the commonly used primer set (Thongprem et al. 2020; Chebbah et al. 2021). PCR assays consisted of a total volume of 15 μL per well, comprising 7.5 μL GoTaq Hot Start Polymerase (Promega), 5.5 μL nuclease‐free water, 0.5 μL forward and reverse primers (concentration 10 pmol/μL) (Table S1) and 1 μL DNA template. A total of 35 COI barcode sequences were also used to establish whether each bedbug specimen was *C. lectularius* or *C. hemipterus* . For samples where COI sequence was not obtained, an RFLP assay was conducted, involving digestion with *MspI* in 10× buffer Tango (Thermoscientific) which selectively cuts the COI amplicon of *C. hemipterus* but not that of *C. lecturalius*.

DNA templates passing this QC stage were then utilised for onward detection of symbionts by PCR assay. Endpoint PCRs were performed targeting *Wolbachia* wsp gene, *Ca*. Tisiphia 16S rRNA & the citrate synthase (gltA) genes and *Symbiopectobacterium* Gyrase B (GyrB) gene (Table S1: https://figshare.com/s/28c4ac95d12fdd361159). All assays used the following PCR conditions: initial denaturation at 95 °C for 5 min, followed by 35 cycles of denaturation (94 °C for 30 s), annealing (Ta °C for 30 s), extension (72 °C for 50 s), and a final extension at 72 °C for 7 min. The annealing temperature varied according to the primers (see Table S1 for primer sequences, Ta and expected product size). All PCR assays included positive (known infected) controls alongside blank (no DNA) negative controls.

PCR products were visualised on 1.5% agarose gels stained with Midori Green Nucleic Acid Staining Solution (Nippon Genetics 41 Europe). From these data, each individual bedbug was allocated to species, and symbiont infection status (present/absent) for each of *Wolbachia*, *Symbiopectobacterium* and *Ca*. Tisiphia. In addition, the mtDNA sequence of the individual was obtained in 35 cases.

Where positive amplification was observed for symbiont infection, the identity of the infection was ascertained by Sanger sequencing for one sample per location per symbiont using the forward primer. Sequences obtained were manually curated and compared to known symbiont sequences for bedbugs by reference to Blastn homology Database (https://blast.ncbi.nlm.nih.gov/Blast.cgi).

### 2.3 Phylogenetic Analyses

The resulting sequence chromatograms were edited and trimmed using Finch TV version 1.4.0 (2004–2006 Geospiza Inc.). Obtained sequences were submitted to GenBank under accession numbers PP887590‐PQ493830‐PQ493831.

The edited sequences were compared against known *Rickettsia* strains in the NCBI database. *Rickettsia bellii* was selected as the outgroup for phylogenetic analysis of both genes (16S rRNA & gltA). All selected *Rickettsia* sequences were then aligned with the new sequences using MAFFT v7.4 (Katoh et al. 2002). ModelFinder (Kalyaanamoorthy et al. 2017) was employed to select the best‐fit models. Maximum likelihood phylogenetic trees were then inferred for both genes using IQ‐TREE (Nguyen et al. 2015) with 1000 rapid ultra fast bootstrap replicates (Minh et al. 2020). TPM2 + F and HKY + F + I models were used for the 16S rRNA and gltA genes, respectively.

The *Symbiopectobacterium* gyrase B dataset was aligned with MAFFTv7.4 (Katoh et al. 2002). Modelfinder was used to ascertain the appropriate model (TIM2 + F + G4). Phylogenetic relatedness was estimated with IQTREE (Nguyen et al. 2015) using 1000 rapid bootstrap replicates (Minh et al. 2020).

### 2.4 Concordance of Bed Bugs' mtDNA Sequences and Their Symbiont Infection Status

The COI sequences for *C. lectularius* above were aligned with MAFFTv7.4 (Katoh et al. 2002). Haplotype diversity indices were calculated for bed bug populations in DnaSP v6.12.03 (Rozas et al. 2003). Subsequently, the haplotypes were extracted, and a haplotype‐spanning network was estimated using the TCS haplotype network algorithm generated in PopART v7 (Leigh, Bryant, and Nakagawa 2015). The symbiont infection status was then mapped to the mtDNA haplotype.

### 2.5 Statistical Analyses

The statistical analyses of symbiont infection status were performed for *C. lecturalis* samples collected from various locations. The data were analysed using the statistical platform R (version 4.3) (R Development Core Team 2022) with the packages “Companion to applied regression(car)” (Fox and Weisberg 2019). To explore associations between endosymbionts, double infection status, and the mtDNA haplotype groups and *C. lecturalis* populations collected from various locations, a Fisher's exact test was performed, with a significance cut‐off set at *p* < 0.05.

## 3 Results

Of the 50 bedbugs tested from 10 locations, 45 were *C. lectularius* and five were identified as *C. hemipterus* .

*Wolbachia* was detected by species‐specific endpoint PCR (wsp gene) in all *C. lectularius* and *C. hemipterus* specimens. *Cimex hemipterus* was not observed to be infected with either of the facultative symbionts. The facultuative symbionts *Symbiopectobacterium* and *Ca*. Tisiphia were detected in *C. lectularius* (Table 2; https://figshare.com/s/96ce0e00318c1caaae11). Infection with these symbionts occurred broadly over space, and individuals carrying all three symbionts were most common. Tests revealed no evidence to reject the null hypothesis that *Symbiopectobacterium* infection frequency occurred evenly between locations (Fisher's exact test, *p >* 0.05). In contrast, heterogeneity among samples was supported for *Ca.* Tisiphia infection, with some samples containing symbionts in all individuals while others had no infected individuals (Fisher's exact test, *p* < 0.05).

**TABLE 2:** Infection status of *C. lectularius* individuals from various areas of Paris. Sy+ = positive for *Symbiopectobacterium*, Sy− = negative or *Symbiopectobacterium*; T+ = positive for *Ca*. Tisiphia, T− = negative for *Ca.* Tisiphia.

Location	Sy+ T+	Sy+ T−	Sy− T+	Sy− T−
District 9 of Paris	5	0	0	0
District 11 of Paris	2	2	1	0
District 18 of Paris	0	1	0	0
District 19 of Paris	4	1	0	0
District 20 of Paris	0	4	0	1
Bobigny	5	0	0	0
La Courneuve	0	5	0	0
Pantin	2	1	1	1
Montreuil	5	0	0	0
Aubervilliers	2	0	1	1
*Total Paris*	25	14	3	3

**FIGURE 1:**
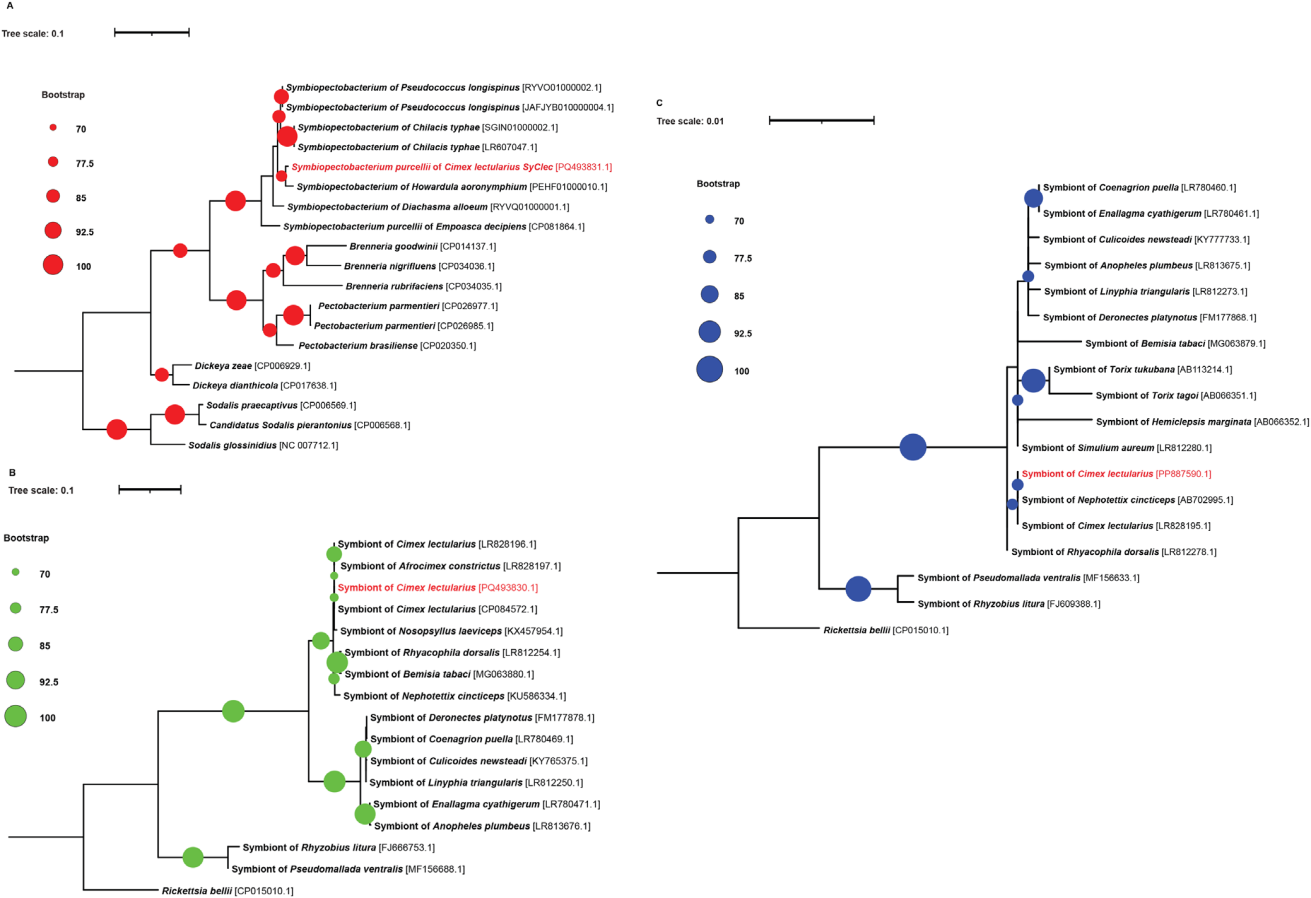
Maximum likelihood phylogenetic trees of facultative symbionts of Parisian *C. lectularius* constructed using gyrB of *Symbiopectobacterium* (A), *gltA* of *Ca*. Tisiphia (B) and 16S rRNA of *Ca*. Tisiphia (C) gene sequences compared to previously recorded congeneric symbiont sequences originating from GenBank. Names given correspond to the host species of the symbiont.

Amplicon sequences obtained for *Ca*. Tisiphia (*gltA*, 16S rRNA) and *Symbiopectobacterium* (*gyrB*) were the same across all individuals examined. The sequences obtained were aligned to *Ca*. Tisiphia and *Symbiopectobacterium* sequences previously described in this species. Phylogenetic analysis compared to other known strains of symbiont clearly clustered the Parisian isolates as monophyletic with the previously recorded strains from bedbugs (Figure 1). The *Wolbachia* sequence from our *C. lectularius* matched 100% with the *Wolbachia* endosymbiont of *C. lectularius* previously reported in this species (KR706518.1).

**FIGURE 2:**
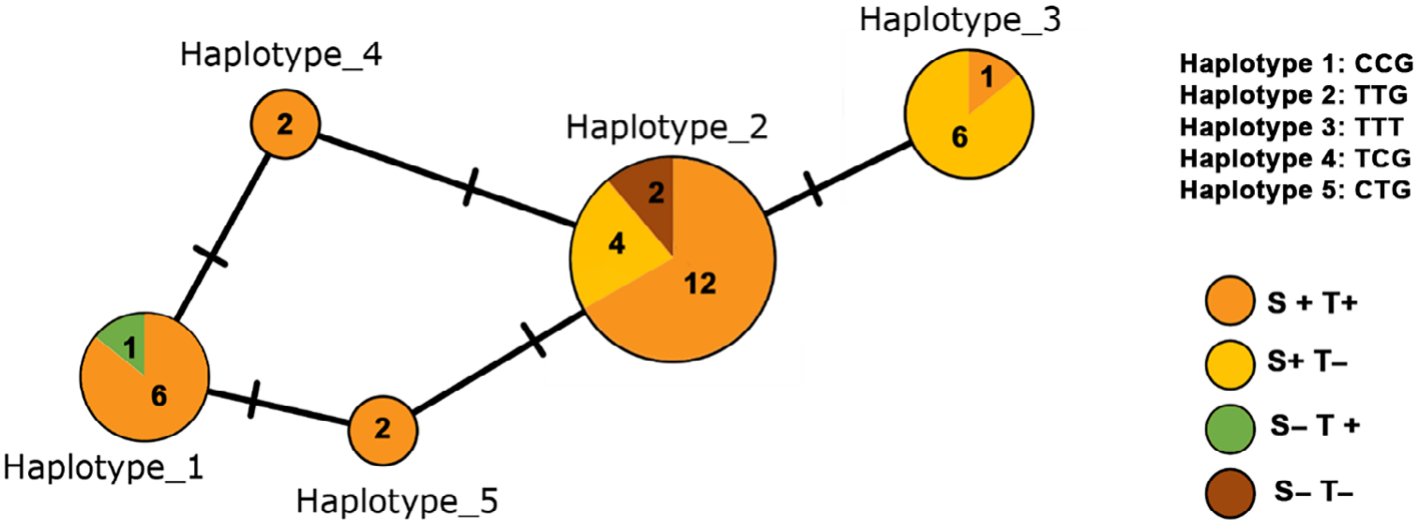
Haplotype spanning network for mtDNA in Parisian *C. lectularius* constructed from 257 bp of COI sequence, partitioned by symbiont infection status. The size of the circle indicates the number of individuals with that haplotype; colours within represent the facultative symbiont infection status of the individuals with that haplotype (Sy+ = positive for *Symbiopectobacterium*, Sy− = negative for *Symbiopectobacterium*; T+ = positive for *Ca*. Tisiphia, T− = negative for *Ca*. Tisiphia). Haplotypes are connected through a spanning network, with the number of nucleotide differences between haplotypes indicated by bars on the line linking the haplotypes.

We then mapped symbiont infection status onto the mtDNA haplotype network (Figure 2). This analysis revealed five mtDNA haplotypes defined by three SNPs across 257 bp of high quality score sequence (> 30) (1.1% of sites segregating in a sample of 35 individuals). The *Symbiopectobacterium*/*Ca*. Tisiphia coinfection was found in all five mtDNA haplotypes obtained; individuals with a single infection (Sy+T−, Sy−T+) or no facultative symbiont infection (Sy−T−) were found in a subset of mtDNA haplotypes.

## 4 Discussion

Bedbugs are among the most common ectoparasites of humans worldwide. While not regarded as biological vectors, they are a serious nuisance pest with a high economic impact. Like many insects, the biology of bedbugs is partly influenced by their symbiotic partners. For *C. lectularius* , these include the obligate symbiont *Wolbachia*, as well as the facultative symbionts *Symbiopectobacterium Sy*Clec and *Ca*. Tisiphia. The presence of facultative symbionts is largely documented in laboratory lineages, while our research focuses on exploring their diversity and frequency of infection in field‐caught bed bug populations and demonstrates their circulation at high frequency in field‐collected samples. However, *Symbiopectobacterium Sy*Clec and *Ca*. Tisiphia were not present in all individuals sampled, corroborating their status as facultative partners.

To this end, we examined the heritable microbiota of bedbugs collected during the Paris outbreak of summer 2023 using a targeted PCR approach. These data represent the first survey of field‐collected bedbugs for the three symbionts. The predominant bedbug species recovered was *C. lectularius* , and all three previously described symbionts from this species were recovered in our samples. Indeed, the majority of individuals collected were coinfected with all three symbionts, *Wolbachia*, *Ca*. Tisiphia and *Symbiopectobacterium*. Other individuals carried either *Wolbachia* alone, or *Wolbachia* along with one of the other two symbiont strains. Theoretical work indicates that where coinfected forms are common, individuals lacking one or more of the infections are expected to be found due to segregational loss (inefficient maternal inheritance) (Frank 1998). The high frequency of triply infected individuals alongside a minority of bedbugs with either just one or neither facultative symbiont makes it likely that the triply infected form occasionally shows segregation of the facultative symbionts, creating doubly infected individuals, and these doubly infected individuals occasionally show segregation of the remaining facultative symbiont resulting in *Wolbachia*‐only infected lines. This model is consistent with data from maintained laboratory lines, where the mix of *Ca*. Tisiphia infected and uninfected individuals led the authors to conclude that *Ca*. Tisiphia showed occasional segregational loss (Thongprem et al. 2020).

The data derive from a single outbreak at a single time, Paris 2023. The sampling within Paris was geographically broad—10 arrondisements—with up to five bed bugs per sample. Thus, whilst our data clearly indicate triple symbiont infected individuals to be most common in this sample, precise symbiont prevalence remains uncertain. Analysis of laboratory lines indicates that these symbionts have a global circulation (Thongprem et al. 2020), but their frequency in other places, and degree of temporal variation, remain unknown and for future study.

In addition, we examined the relationship between symbiont status and mitochondrial variation. Mitochondria are also maternally inherited, such that the dynamics of symbionts impact that of mitochondria and *vice versa* (Galtier et al. 2009; Fenton, Camus, and Hurst 2021). These data recovered five mtDNA haplotypes from the COI amplicon sequence and 1.1% of mtDNA sites are segregating within our sample of 35 individuals. The diversity of mtDNA types found indicates there has not been a recent selective sweep of either mtDNA or symbionts, as recent selective sweeps of symbionts or mtDNA are associated with highly reduced mtDNA variation, and commonly a single circulating mtDNA haplotype. Our data, in contrast, lead us to infer that the symbionts have likely been circulating in the species for some time. This conclusion is consistent with *Symbiopectobacterium Sy*Clec and *Ca*. Tisiphia being recorded from *C. lectularius* across continents in previous work (Hypša and Aksoy 1997; Thongprem et al. 2020; Pilgrim et al. 2021; Davison et al. 2022).

Past work has emphasised the functional significance of *Wolbachia* to bedbugs and indicated the presence of *Ca*. Tisiphia and *Symbiopectobacterium Sy*Clec. Our study provides population‐level data for the frequency of these facultative partners in the Parisian outbreak, and indicates that bedbugs were most commonly triply infected with symbionts. Furthermore, the mtDNA data indicate that the symbionts have been present for a sufficient evolutionary time that they are associated with diverse mtDNA haplotypes. What is not clear is their functional significance. Their persistence over time and their circulation at high frequency, combined with evidence that they do not cause reproductive parasitism (Thongprem et al. 2020), make it most parsimonious to consider them as partners of unknown (but significant) benefit.

## Author Contributions

**Naciye Sena Cagatay:** conceptualization, data curation, formal analysis, funding acquisition, investigation, writing – original draft, methodology, validation, visualization, writing – review and editing, software. **Mohammad Akhoundi:** conceptualization, investigation, project administration, supervision, resources, writing – review and editing, writing – original draft. **Arezki Izri:** investigation, writing – review and editing. **Sophie Brun:** investigation, writing – review and editing. **Gregory D. D. Hurst:** writing – review and editing, writing – original draft, project administration, resources, supervision, methodology, formal analysis, conceptualization.

## Conflicts of Interest

The authors declare no conflicts of interest.

## Supporting information


**Table S1.** Symbiont presence and mitochondrial haplotype of bedbug individuals collected in Paris 2023.

## Data Availability

The raw data underpinning Table S1 (https://figshare.com/s/28c4ac95d12fdd361159) and raw data (https://figshare.com/s/96ce0e00318c1caaae11) can be accessed at figshare. Marker gene sequences are available under accessions PQ49383.1‐PQ493830.1‐PP887590.1‐PP886873‐PP886877.
